# Refinements of the phase adjustment task (PAT 2.0)

**DOI:** 10.3389/fpsyg.2026.1677186

**Published:** 2026-05-29

**Authors:** Ren Palmer, Jennifer Murphy, Jonathan M. Bird, Rosie Donaghy, Tom Piercy, Kiera L. Adams, Connor T. Keating, Rachel Tam, David Plans, Davide Morelli, Adam Cunningham, Geoffrey Bird

**Affiliations:** 1 School of Psychology, University of Surrey, Guildford, United Kingdom; 2 Department of Targeted Intervention, University College London, London, United Kingdom; 3 Department of Experimental Psychology, University of Oxford, Oxford, United Kingdom; 4 Department of Psychology, Royal Holloway University of London, Egham, United Kingdom; 5 Department of Engineering Science, Institute of Biomedical Engineering, University of Oxford, Oxford, United Kingdom; 6 Independent Researcher, London, United Kingdom; 7 Centre for Research in Autism and Education, Institute of Education, University College London, London, United Kingdom; 8 European Research College London, London, United Kingdom; 9 European Research University, Ostrava, Czechia

**Keywords:** cardiac interoception, cardiac interoceptive accuracy, interoception, interoceptive accuracy, interoceptive awareness

## Abstract

In 2021 a new measure for the assessment of cardiac interoceptive accuracy—the Phase Adjustment Task (PAT)—was developed that overcomes several limitations of existing methods and can be administered using a smartphone application. In this report, we describe several refinements to the PAT. These include: (1) triggering tones from the detection of the heartbeat via the smartphone camera, rather than using an algorithm for predicting the occurrence of the next heartbeat; (2) technical amendments to enable implementation on iPhones with multiple cameras; (3) changes to instructions and (4) changes to the collection of confidence ratings to improve participant understanding and the utility of confidence ratings for interpretation of results; (5) the introduction of new practice trials to improve clarity; (6) changes to the analysis approach to identify cardiac phase-based heartbeat perceivers; (7) a review of the use of continuous scores; (8) a reanalysis of data comparing PAT performance in supervised laboratory settings and unsupervised remote settings using the new analysis method, and (9) the implementation of additional measures to discourage participants from checking their pulse. In this paper we outline our justification for these changes and provide details of where researchers can access the materials required for implementing the PAT and analyzing data. Finally, we provide further recommendations for implementation.

## 1 Introduction

In recent years there has been an increased focus on the measurement of cardiac interoceptive accuracy, the perception of internal bodily signals arising from the heart (Brewer et al., 2021). Such increased focus has been driven by concerns regarding the validity of commonly used measures of cardiac interoceptive accuracy (Desmedt et al., 2023). For example, the heartbeat counting task (HCT; Dale and Anderson, 1978; Schandry, 1981), one of the most commonly used measures of cardiac interoceptive accuracy, suffers from the flaw that individuals who know their heart rate can falsely appear able to perceive their heartbeats (e.g., Desmedt et al., 2018; Murphy et al., 2018). In addition, the heartbeat detection task (HDT; first proposed by Whitehead et al., 1977), also commonly used, has the flaw that it assumes that all participants perceive their heartbeat at the same point in the cardiac cycle—an assumption shown to be invalid (e.g., Yates et al., 1985; Ring and Brener, 1992).

These concerns have given rise to the development of several new measures. The Phase Adjustment Task (PAT; Plans et al., 2021) has been described as one of the most promising of the new measures of cardiac interoceptive accuracy (Desmedt et al., 2023). In this task, tones are presented at a rate matching the participant’s heart rate, but out of phase with heartbeats. Participants are asked to turn a virtual dial to adjust the phase relationship between heartbeats and tones until they believe the tones to be synchronous with their heartbeat. As the starting phase is random across trials, the consistency of the participant’s response is taken as a measure of accuracy (as participants who are unable to feel their heartbeats should choose phase relationships at random across trials). Bayesian analyses were used to classify participants as interoceptive, non-interoceptive or unclassified, reflecting the degree of certainty surrounding evidence for cardiac perception at various thresholds. The PAT was originally programmed to be administered via a smartphone application (of course, it can also be implemented in a lab setting), providing opportunities to increase the size and diversity of samples in interoception research. Although the PAT overcomes several limitations of existing measures (chiefly that knowledge of heartrate is insufficient to provide above-chance performance in the absence of the ability to detect heartbeats, and no assumption is made about when in the cardiac cycle heartbeats are perceived; for discussion see Plans et al., 2021; Desmedt et al., 2023), the specific way it was implemented and analyzed could be improved, motivating the development of the updated implementation reported here (PAT 2.0). In brief (discussed in more detail throughout), issues existed with the presentation of tones relative to heartbeats, camera selection on multi-camera iPhones, task instructions and the collection of confidence ratings, and (in common with all other tasks to our knowledge) an analysis procedure which was unable to identify interoceptive individuals who perceived their heartbeat at a consistent phase of their cardiac cycle (rather than a consistent delay from the preceding heartbeat). The steps taken to address these issues are detailed in the following, with recommendations for implementation aimed at further optimizing the measurement of cardiac interoceptive accuracy. Code for implementing the revised tasks described below, alongside analysis scripts, can be found at https://osf.io/fp5sq/ and https://github.com/Interolab/Interoception.

## 2 Methods

### 2.1 Refinements

#### 2.1.1 Heartbeat detection

In the original version of the task, detected heartbeats were not used to determine when tones occurred. Instead, the onset of tones was governed by the predicted occurrence of heartbeats derived from beat-to-beat intervals recorded every 3 s throughout the task. However, given that the accuracy of this prediction could vary both across participants and within participants on different testing occasions due to differences in heart rate variability, it is more desirable to use detected heartbeats to govern tone onset. The original decision to base tones on predicted heartbeats was due to the unfounded concern that participants may perceive their heartbeat before it was recorded at the finger due to the delay between the contraction of the heart and the arrival of the pulse at the finger. As analysis of the PAT focuses on consistency, however, it is not problematic for participants to perceive their heartbeat before the pulse wave is recorded at the finger. Even when a participant feels their heartbeat before it is detected at the finger, they would still be able to demonstrate perfect consistency by selecting the same phase relationship between tones and detected heartbeats across trials. As such, in PAT 2.0, detection of the heartbeat at the finger governs tone occurrence. This ensures that any potential differences in the accuracy of the prediction of heartbeats, within or across participants, cannot influence scores. As part of this change, the range of the dial is now set by the greater of either the running average period between heartbeats or last recorded period between heartbeats (“instant period” in the analysis script). This ensures that the dial always spans a physiologically plausible interval and reduces distortions arising from anomalous heartbeat period readings. The analysis script has been updated accordingly such that the dial range period used in the analysis script matches the new approach to setting the dial range in the task. However, similarity calculations (described in section 2.1.6.3) continue to use the last recorded heartbeat period, as these reflect heartbeat dynamics when participants submit their delay selection.

#### 2.1.2 Camera selection

An issue was identified with the implementation of the PAT task on iPhone models that have multiple camera lenses—the iPhone automatically switched the active camera lens, and this interfered with data collection. We have now implemented edits to the code to ensure that one camera lens is selected by the app and used throughout the task, so that the task can be completed by participants using any existing iPhone model running iOS 15 and above (providing operability with 94% of iPhones in current use; Sherif, 2024).

#### 2.1.3 Changes to participant instructions and procedure

**FIGURE 1:**
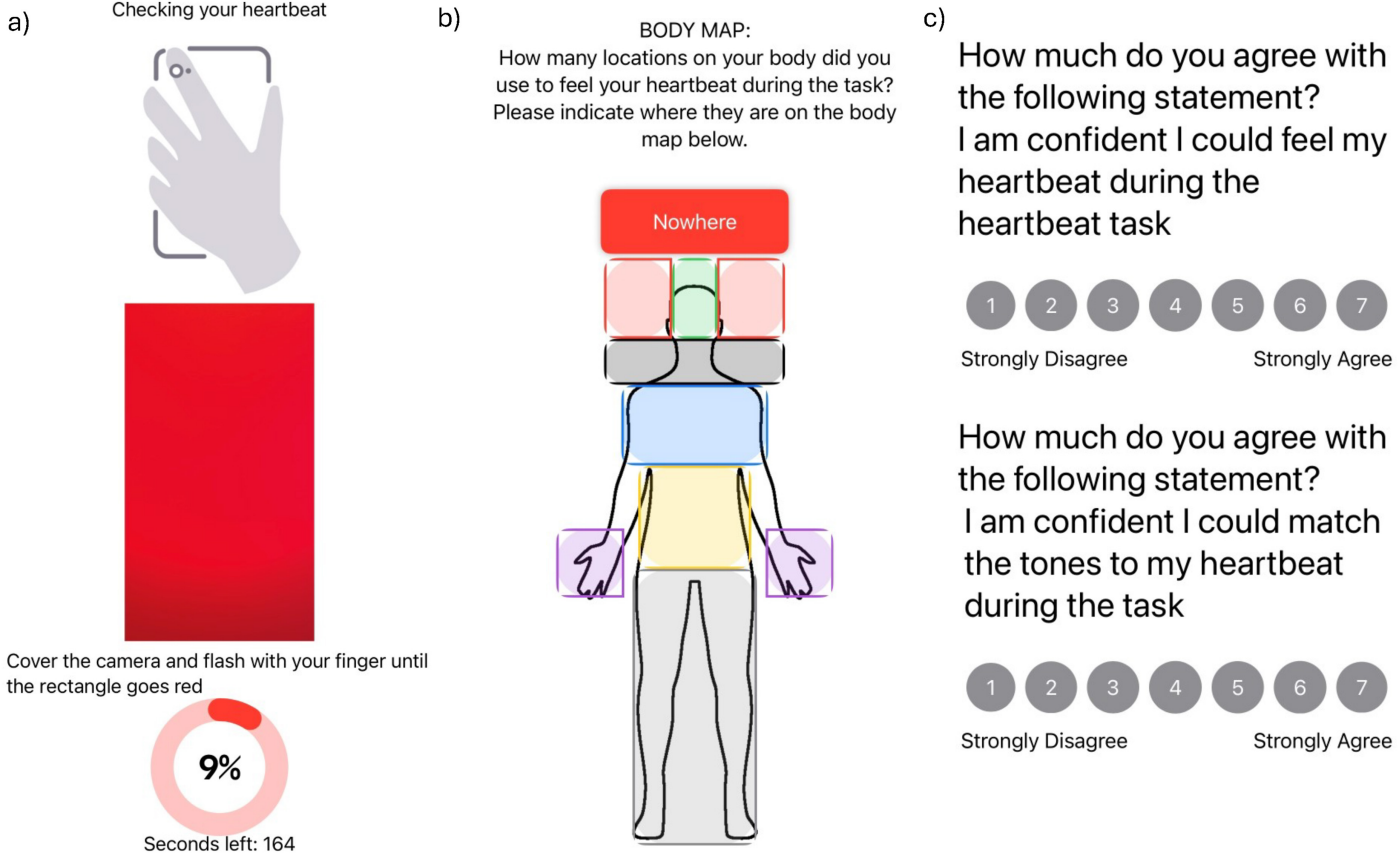
Visual depiction of testing screens. **(a)** An example of the visual depiction of camera capture ensuring correct finger placement over the camera and flash. Participants are instructed to “make the rectangle red” by placing their finger over the camera and flash. **(b)** An example of the body maps. **(c)** An example of the confidence ratings collected at the end of the task.

To increase clarity for participants, we implemented various changes to the instructions and instruction procedure. Full details of changes to the instructions can be found in Supplementary material. Central changes to the instructions included: (1) a description of the cheat-avoidance mechanism (described in section 4.1); (2) changes to the procedure for incorrect finger placement; and (3) changes to the collection of confidence ratings and the location from which heartbeats are perceived. In terms of finger placement, in the original instantiation of the PAT, when the recording of heartbeats was disrupted (e.g., the participant’s finger was not adequately covering the smartphone camera and flash) participants were presented with a prompt asking them to readjust the position of their finger. To reduce disruption during the task, participants are now presented with a visualization of the camera recording at the start of the study and are asked to “make the rectangle red” by covering the camera and flash with their finger. This visualization disappears as soon as they are in the correct position, and participants are presented with the visualization during the task whenever their finger does not adequately cover the smartphone camera and flash (determined by analyzing the color feed against various thresholds; see Figure 1a).

#### 2.1.4 Confidence ratings and body maps

Trial-by-trial confidence ratings have been removed as we could not conceive of how they would be useful (there is no correct answer in the PAT, so the usual confidence-accuracy correlation across trials cannot be calculated to assess metacognitive interoceptive insight, and these measures are likely of little validity anyway with low trial numbers). In PAT 2.0, we now record global confidence at the end of the task where participants are asked to rate on a 7-point Likert scale ranging from Strongly Disagree to Strongly Agree, their agreement with the following statements “I am confident I could feel my heartbeat during the heartbeat task.” and “I am confident I could match the tones to my heartbeat during the task.” These questions allow separation of confidence related to the perception of heartbeats, and confidence in one’s ability to perform the task. The same questions are also used in the screener task (see section 2.1.5) for completeness, though refer to tones rather than heartbeats (see Figure 1c).

Body maps (see Figure 1b) are now shown once at the start and once at the end of the task, as opposed to every 5 trials as per the original PAT. Participants are now explicitly told to pick one location to perceive their heartbeats and to stick to that one location during the whole task. The first body map asks participants where they feel their heartbeat most clearly. The second asks how many locations they used to feel their heartbeat during the task. Reducing the amount of body maps throughout the task reduces the overall completion time (see Figure 1b).

#### 2.1.5 Screener task

In the original study describing the PAT (Plans et al., 2021), a structurally identical screener task was included in Study 2 in which participants were asked to synchronize two tones, with one of these tones (unbeknownst to participants) representing their heartbeat. The aim of this screener task was to familiarize participants with the procedure, including the cheat-avoidance mechanism, and the code for this task is available at https://github.com/Interolab/Interoception. In recent studies using this task (i.e., Spooner et al., 2024a,b) the screener is used to select participants to progress to the PAT, setting a minimum consistency score of ∼.42. This consistency score cut off value is taken from the probability function estimating the distribution of interoceptive and non-interoceptive participants from Plans et al. (2021) where the probability of being non-interoceptive is approximately 0 for a consistency score of 0.42 with 95% confidence. However, data from a recent project suggests that a more conservative score may be a more appropriate cut off for selecting participants given that matching two tones is far easier than matching a tone to one’s heartbeat (Plans et al., under review). For future use, we would recommend that users score the screener task using the updated scoring approach (described in section 2.1.6 below) and include only individuals who demonstrate above chance performance. Regardless of the exact cut off used, however, the use of the screener task alongside the PAT is invaluable, as it enables it to be determined whether individuals who score poorly on the PAT do so because of poor understanding of instructions, low motivation/attention, poor timing, wider cognitive impairment etc. (in which case they should also score poorly on the screener task), or whether they just cannot perceive their heartbeats (in which case they should perform well on the screener, but poorly on the PAT). For those choosing not to use the screener task, in PAT 2.0 there are now two practice trials where participants are asked to synchronize two tones, followed by two practice trials where participants are asked to synchronize a tone to their heartbeat, to increase task clarity.

#### 2.1.6 Task scoring

In terms of task scoring, we have made a number of revisions to the analysis code to better estimate the likelihood that participant behavior represents non-random responding.

##### 2.1.6.1 Dial range calculations

As described above (see section 2.1.1), to ensure that the dial range represents a physiologically plausible range, the greater of the average period between heartbeats for each trial and the last recorded period between heartbeats is used to set the dial range. However, similarity calculations continue to use the last recorded heartbeat period, as these reflect heartbeat dynamics when participants submit their delay selection. As the new script uses the final position median inter-beat interval (IBI, used in similarity calculations; see 2.1.6.1), on rare occasions (2.1% of trials) the delay selected may exceed the dial range. Where delays exceed the final position median inter-beat interval, delays are wrapped by subtracting this median IBI from the delay so that all delays remain within the bounds of the period used for analysis.

##### 2.1.6.2 Data cleaning

The data now undergo more rigorous cleaning based on heart rate metrics within each trial. The current script excludes trials where heart rate falls outside of physiologically plausible limits (set as 40–120 bpm). These parameters are appropriate when participants are at rest, and can be adjusted for other situations (e.g., exercise).

We have also introduced more rigorous cleaning of the 3-min baseline period. To ensure physiological plausibility and minimize the influence of artifacts, IBIs are screened using predefined lower and upper bounds (0.5–1.5 s), corresponding to the range of 40-120 bpm noted above. For each participant, the longest continuous segment of IBIs within this valid range is identified. Participants whose longest valid segment does not meet a minimum length criterion of 30 s (adjustable in the analysis scripts) are flagged as having insufficient baseline data, as heart rate variability (HRV) indices can be unreliable when derived from short recordings (Bourdillon et al., 2017: cf. Munoz et al., 2015).

In addition, the code now also implements additional cleaning to remove invalid trials (e.g., triggering of the cheat avoidance mechanism; see section 4.1) and participants where the task was interrupted (e.g., due to receiving a call or message during the session).

##### 2.1.6.3 Similarity score formula

When previously analyzing PAT data, prior to computing consistency scores we mapped each delay to an arc length of 2π*d*/*p* (where *d* = delay and *p* = heartbeat period) on a unitary circumference. However, simulations suggested that the use of arcs introduced a bias with extreme heart rates. In PAT 2.0, although the same fundamental formula is used, $c\,o\,n\,s\,i\,s\,t\,e\,n\,c\,y\,\left(d,p\right)\,\frac{1}{n}\,m\,o\,d\,\left(\sum_{j\,1}^{n}e^{i\,2\,\pi \,\frac{d_{j}}{p_{j}}}\right)$, we now treat each delay as a phase angle (2π*d*/*p*) on a unitary circumference, rather than as an arc length. In practice, this means delays are expressed as a fraction of the cardiac cycle (i.e., a periodic angle on a unit circle). This simplifies the analysis and avoids the potential bias at extremes of heart rate under the previous method.

Furthermore, our previous analysis approach examined the consistency of participants’ selected delays over trials. However, where inter-beat-intervals over trials are variable, this approach would likely fail to capture interoceptive individuals who consistently report feeling their heartbeat at a certain phase of their cardiac cycle, rather than an absolute delay after r wave onset. The use of a single approach (phase or delay) may fail to accurately identify some interoceptive individuals, but to our knowledge no current analysis approach addresses these two possibilities (Desmedt et al., 2023). To account for this, PAT 2.0 now introduces a simplified scoring framework combining two complementary methods.

##### 2.1.6.4 Phase-based consistency scoring for phase-consistency

To capture individuals who are selecting the same phase of their cardiac cycle over trials, our analysis first applies the formula outlined above to calculate consistency scores for each participant. To avoid the potential influence of bad recordings on the heartbeat period used for analyses, PAT 2.0 now uses the median inter-beat-interval (IBI) from the final dial position (where participants kept their finger on the same dial position +/-5 degrees prior to submitting their response) as the heartbeat period (*p*) rather than the last IBI recorded. Use of a trial-by-trial heartbeat period allows for detection of participants who align their responses to a proportion of their cardiac cycle, even when IBI changes across trials. A group-level cutoff for classification as interoceptive is defined from 100,000 simulations using randomly generated *p-*values (between 0.5 and 1.5 s) and randomly generated delay sequences (within ± 1/2 *p*). The 95th percentile of this distribution serves as the threshold above which a participant is classified as interoceptive.

**FIGURE 2:**
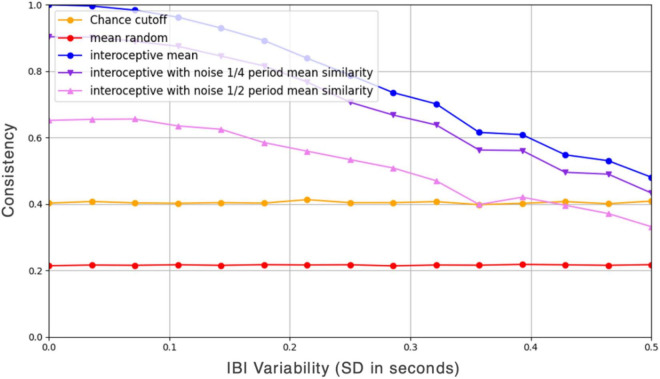
Phase consistency [i.e., consistency of delays as a function of Inter-Beat-Interval (IBI)] as a function of IBI variability over trials (here expressed as the standard deviation of IBIs in seconds). The red line is the mean consistency score for a group of 100 simulated random responders, with the orange line indicating the 95th percentile of this random distribution and therefore a cutoff score above which there is less than a 5% chance that the consistency score would be generated by a random responder. The blue line shows the scores of perfectly consistent participants, while the darker and lighter purple lines show the consistency scores expected for interoceptive participants with 1/4 and a 1/2 period of added noise, respectively.

**FIGURE 3:**
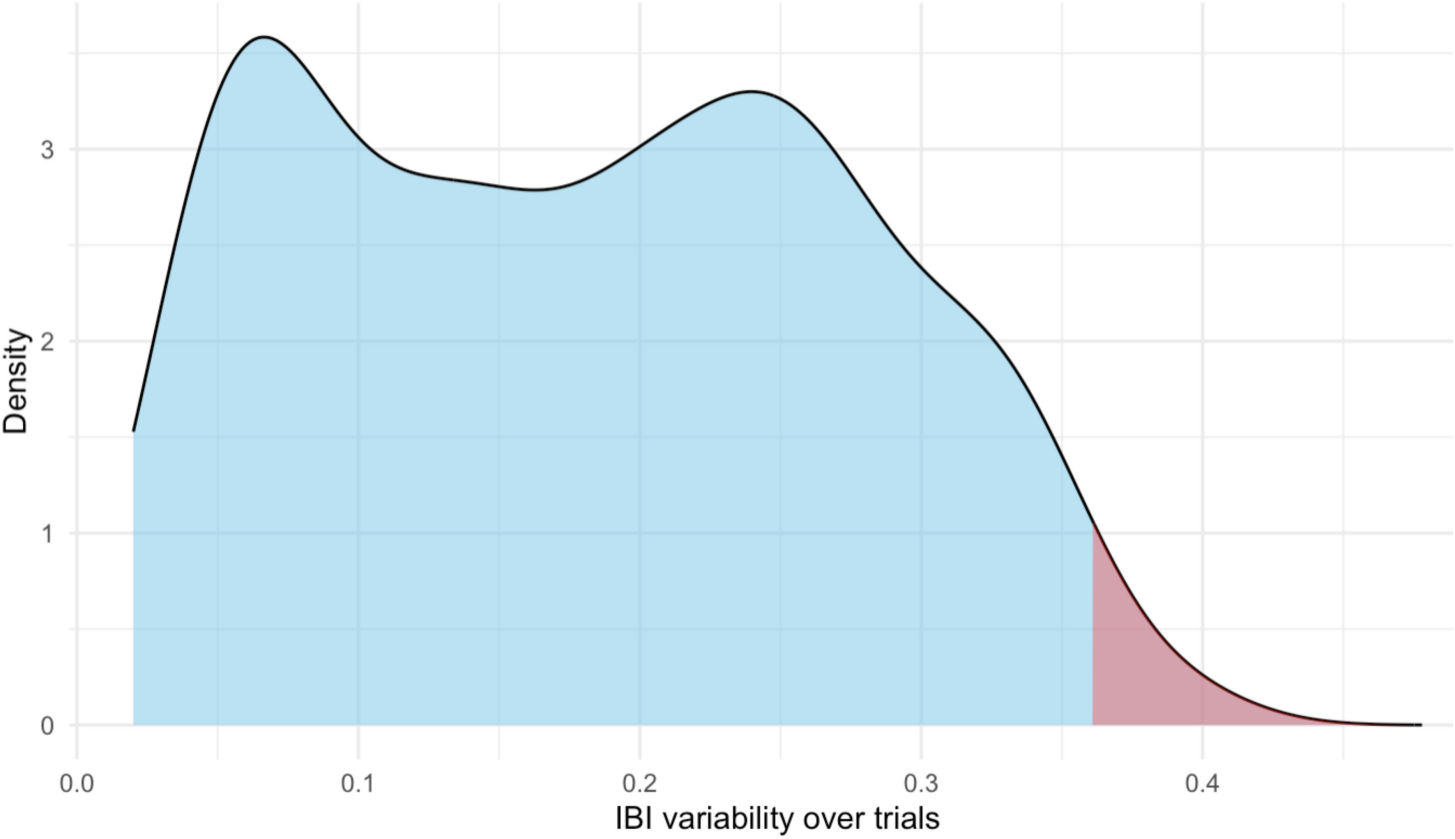
Density plot of variable heart rate over trials (here expressed as the standard deviation of inter-beat-intervals in seconds) in 693 completions of the PAT. The area shaded in red represents participants whose IBI variability over trials falls beyond a threshold of 0.36 (*N* = 12).

One challenge to the use of phase-based consistency is high levels of IBI variability over trials.^1^ However, simulations using this new phase-based consistency method indicate that concerns regarding high levels of IBI variability over trials affecting classification scores are unfounded. As can be seen from Figure 2, even at levels of variability far beyond what is likely for most participants there is still a clear difference between consistency scores expected for interoceptive participants (even with very high levels of noise), and participants responding at random. Real data from 693 PAT completions (*N* = 531 participants across multiple published and unpublished datasets) suggests that IBI variability over trials is well within the reasonable limits for classification as demonstrated by the simulation below (Figure 2). Of these real participants, only 1.7% would fall beyond a threshold that may give cause for concern for an interoceptive participant with incredibly high levels of noise (>0.36 IBI variability over trials; Figure 3).

##### 2.1.6.5 Fixed-period consistency scoring for delay-consistency

The second approach, to identify those participants selecting consistent delays over trials (regardless of heartbeat period), utilizes fixed-period consistency scoring. In this approach, delays are still expressed as a periodic function of the heartbeat period (angle), but the period is fixed using a single, participant-specific heartbeat period for all trials (computed as the median IBI of all median IBIs from the final dial position over trials). We then use the same consistency formula described above, but with a constant *p* (period) value.

**FIGURE 4:**
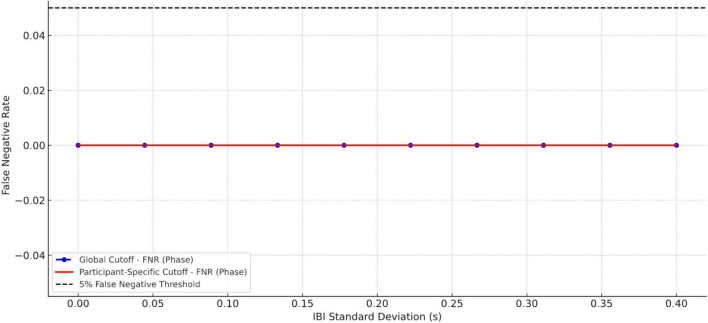
False negative rates for phase-based consistency as a function of IBI variability over trials (here expressed as the standard deviation of IBIs in seconds). The red line is the participant-specific false negative rate (FNR) using participant-specific cutoffs, whilst the blue line represents the FNR using a global cutoff (both overlaid in this graph). The black dotted line indicates the 5% FNR threshold of 0.36.

**FIGURE 5:**
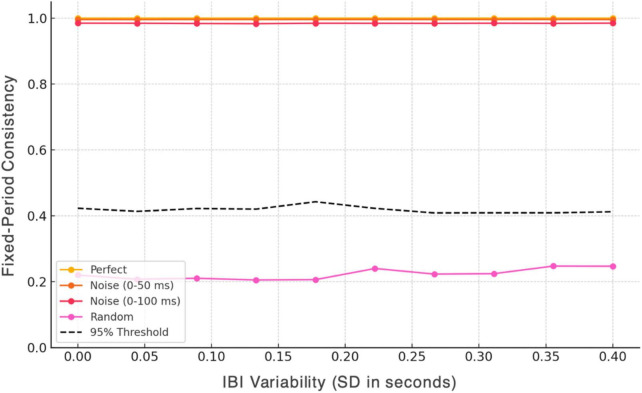
Fixed-period consistency as a function of IBI variability over trials (here expressed as the standard deviation of IBIs in seconds) with heart rate set at 60 bpm. The pink line is the mean consistency score for a group of 100 simulated random responders, with the black dotted line indicating the 95th percentile of this random distribution and therefore a cutoff score above which there is less than a 5% chance that the consistency score would be generated by a random responder. The orange line shows the scores of perfectly consistent participants, while the lighter and darker red lines show the consistency scores expected for interoceptive participants with up to 50 and 100 ms of added noise, respectively.

**FIGURE 6:**
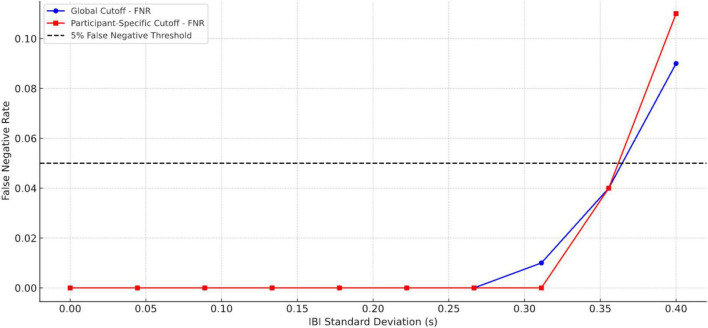
False negative rates for fixed-period consistency as a function of IBI variability over trials (here expressed as the standard deviation of IBIs in seconds). The red line is the participant-specific false negative rate (FNR) using participant-specific cutoffs, whilst the blue line represents the FNR using a global cutoff (both overlaid in this graph). The black dotted line indicates the 5% FNR threshold of 0.36.

For fixed-period scoring, an individualized cut-off score is required. This is because unlike phase-based scoring, where chance thresholds are unaffected by IBI variability over trials (see Figures 2, 4), fixed-period thresholds are slightly influenced by IBI variability. Participant-specific thresholds are therefore more appropriate (see Figures 5, 6). Note, under both scoring approaches there is no longer an unclassified category.

To derive an individualized cutoff, we perform 10,000 simulations per participant by generating random delay sequences (within ± 1/2 *p*) and computing their consistency scores using the participant’s *p*. The 95th percentile of this distribution serves as the threshold above which this specific participant is classified as interoceptive under this criterion. Participants whose consistency exceeds their individual cutoff are labeled as interoceptive.

##### 2.1.6.6 Combined classification

Overall classification as interoceptive or non-interoceptive is now determined according to whether a participant’s score is above-chance using either of the above scoring systems. In the new PAT 2.0 analysis, we examine whether participant responses are non-random using both the fixed-period approach and the phase-based approach. This combined system maximizes sensitivity and captures two distinct interoceptive profiles; phase-based (consistent proportional phase selection within individual trials) and delay-based (consistent absolute delay selection). Those meeting either criterion are considered interoceptive under the new dual-method framework, providing a comprehensive and assumption-free characterization of cardiac interoceptive accuracy. Notably, evidence of above-chance performance using both systems should not be taken as evidence of greater interoceptive ability. Indeed, such a scenario likely occurs due to high IBI stability over trials making phase-based and delay-based perception appear equivalent, meaning that the type of perception (delay or phase) cannot be distinguished.

##### 2.1.6.7 The use of continuous scores

One key difference between our novel analysis approach and our previous approach (see Plans et al., 2021) is that continuous consistency scores can no longer be used. Indeed, whilst originally a Bayesian approach was used to classify participants according to the likelihood that they were interoceptive or not, continuous consistency scores were available (Plans et al., 2021; Murphy and Bird, 2024). Although we have always argued that classification (can a participant perceive their heartbeats or not) provides a more relevant metric to cardiac interoceptive accuracy than a continuous score (reflecting heartbeat perception plus task performance), our new analysis approach makes the recommendation of classification scores a requirement. This is because raw consistency scores used to capture phase-based and delay-based consistency are not comparable, and both delay-based and phase-based consistency metrics are also influenced by IBI variability over trials (although this influence is minimal for delay-based consistency scores). Indeed, despite the simulations showing that IBI variability over trials does not impact classification scores for either method, it does show that maximum consistency scores will be impacted by variable IBI over trials, underscoring that continuous scores should not be used to compare participants (at least with different IBI variability over trials). Whilst this prevents examination of individual differences for data collected on one occasion (beyond interoceptive or not), we do not believe this to be a limitation. Indeed, we have argued that rather than whether participants can perceive their heartbeat on one occasion, the number of situations in which an individual can perceive their heartbeat at above chance levels (e.g., during rest, different body positions, under perturbation conditions such as exercise) may be a more useful individual difference measure (Murphy and Bird, 2024) that can be achieved by examining classification scores across time and situations. Thus, being judged as “non-interoceptive” at one time-point, under one set of (internal and external) conditions, for one domain (cardiac), should not be used to make clinical claims about interoception across domains and time/situations.

Additionally, it is worth noting that despite the above evidence suggesting highly variable heart rate over trials does not significantly impact the classification of individuals as interoceptive or non-interoceptive using either method, it is worth exercising caution when taking samples where heart rate is expected to vary substantially over the course of the task. Although data from our sample suggests very low levels of heart rate change over trials, all data used in our sample was collected when participants were at rest (and therefore had relatively stable heart rates). Subsequently, it is recommended that in the event the PAT 2.0 is used for data collection when the participant is not at rest (for example, examining interoceptive accuracy after physical exercise), an extended approach is taken whereby trial numbers are increased and only trials with stable variability in heart rate over trials are analyzed.

### 2.2 Reanalysis of Spooner et al. (2024a) using the new scoring method

Here we present a reanalysis of data comparing scores on the PAT from participants completing the task either supervised, in the laboratory, or unsupervised, remotely. The reanalysis utilizes the new scoring method described in section 2.1.6. As no participants had IBI variability exceeding a threshold that may give cause for concern (see section 2.1.6), the full sample was retained.

#### 2.2.1 Participants and procedure

For full details of the procedure and demographics please see Spooner et al. (2024a). We compare PAT performance from completion in the laboratory to remote completion for the full sample of participants, and for those who had previously passed the Screener Task described in section 2.1.5.

## 3 Results

### 3.1 Full sample

In the full sample, the proportion of participants classified as interoceptive who completed the PAT in the laboratory vs. remote did not significantly differ [*χ^2^*(1, *N* = 200) = 0.03, *p* = 0.866; Table 1]. Examination of demographic data revealed that laboratory vs. remote participants significantly differed in age (see Table 2 for full results). To ensure that differences in interoceptive accuracy were not driven by these differences, groups were matched following the same protocol as in Spooner et al. (2024a). To match groups, we excluded participants aged 30 years and over from the remote cohort and participants under 19 years from the laboratory cohort, as well as one participant from the laboratory cohort who was an outlier in terms of age (aged 63 years). After matching groups, there was still no significant difference in the proportion of laboratory and remote participants classified as interoceptive [*χ^2^*(1, *N* = 116) = 0.93, *p* = .335; Table 1].

**TABLE 1:** New combined phase-based and fixed-period analysis classifications for the (a) full unscreened sample, (b) unscreened sample matched for age, (c) unscreened sample matched for age, for whom valid baseline data was available (d) screened sample, and (e) screened sample matched for age (f) screened sample matched for age, for whom valid baseline data was available.

Sample	Laboratory			Remote			χ ^2^	*p*
	Interoceptive	Non-interoceptive	% interoceptive	Interoceptive	Non-interoceptive	% interoceptive		
(a)	16	70	22.86	19	95	20.00	0.029	0.866
(b)	8	50	16.00	13	45	28.89	0.930	0.335
(c)	6	31	19.35	12	36	33.33	0.511	0.475
(d)	16	65	24.62	15	73	20.55	0.065	0.798
(e)	8	46	17.39	9	33	27.27	0.328	0.567
(f)	6	28	21.42	9	25	36.0	0.342	0.559

**TABLE 2:** Demographic, engagement metrics and heart rate data for laboratory and remote participants in the full and matched samples (with and without baseline data).

Metric	Unscreened sample unmatched for age				Unscreened sample matched for age				Unscreened sample matched for age with baseline			
	Laboratory	Remote	*U*	*p*	Laboratory	Remote	*U*	*p*	Laboratory	Remote	*U*	*p*
	M(SD)	M(SD)			M(SD)	M(SD)			M(SD)	M(SD)		
Total time on task (s)	363.15 (181.76)	408.19 (223.70)	4396.5	0.213	377.59 (196.65)	405.52 (202.61)	1531.5	0.408	365.84 (151.39)	423.58 (213.47)	775.0	0.320
Mean time per trial (s)	21.36 (10.69)	24.01 (13.16)	4396.5	0.213	22.21 (11.57)	23.85 (11.92)	1531.5	0.408	21.52 (8.91)	24.92 (12.56)	775.0	0.320
Mean engagement^1^	26.31 (15.20)	27.77 (16.21)	4642.0	0.522	27.40 (16.70)	27.33 (14.07)	1600.5	0.655	25.66 (12.13)	28.49 (14.97)	798.5	0.430
N of valid trials	19.28 (0.84)	19.05 (1.03)	5399.0	0.189	19.26 (0.91)	19.00 (1.08)	1885.5	0.228	19.19 (0.91)	19.04 (1.07)	938.5	0.634
Age	23.10 (7.71)	31.06 (9.53)	1949.5	**<0.001**	24.78 (6.74)	23.62 (2.74)	1671.5	0.956	25.19 (7.20)	23.69 (2.79)	926.0	0.738
Resting heart rate (bpm)	–	–	–	**–**	–	–	–	–	74.82 (12.36)	73.52 (10.39)	929.0	0.721
Heart rate variability (SDNN)	–	–	–	**–**	–	–	–	–	122.40 (76.05)	89.35 (51.32)	1127.0	**0.034**
Heart rate variability (RMSSD)	–	–	–	**–**	–	–	–	–	90.21 (44.21)	83.15 (40.54)	968.0	0.483
Heart rate variability (pNN50)	–	–	–	**–**	–	–	–	–	41.94 (16.64)	40.89 (19.64)	938.0	0.661

Bold text denotes significance.

To examine the effects of HR and HRV metrics, we restricted the matched sample to those for whom we obtained valid baseline heart rate data. In this matched sample with valid baseline data, we observed no significant difference in the proportion of laboratory and remote participants classified as interoceptive [*χ^2^*(1, *N* = 85) = 0.51, *p* = .475; Table 1]. As significant differences were observed between laboratory and online participants for HRV (SDNN), this variable was subsequently regressed out from the classifications using a logistic model. In this model, PAT scores were the dependent variable, with completion type (laboratory vs. remote) and HRV (SDNN) entered as predictors. Again, this revealed no significant differences in classification scores for laboratory vs. remote participants [β(81) = −0.65, *p* = 0.518].

### 3.2 Screened sample

We repeated the previous analyses after restricting the sample to individuals who had completed the screener task and passed with a score above chance (note the screener task was also scored using the new analysis method described in section 2.1.6). This included 169 individuals (*N*remote = 88). In this sample, the proportion of laboratory vs. remote participants classified as interoceptive did not significantly differ [χ^2^(1, *N* = 169) = 0.07, *p* = 0.798; Table 1]. No differences in engagement metrics were found between groups (*p*s > 0.05), but differences in HR, HRV (SDNN) and age were observed (*p*s < 0.05; see Table 3). After following the aforementioned matching process, there was still no significant difference between laboratory and remote participants [χ^2^(1, *N* = 96) = 0.33, *p* = 0.567; Table 1]. After restricting the matched sample only to individuals for whom valid baseline data was available, there was again no significant difference between remote and laboratory participants [χ^2^(1, *N* = 68) = 0.34, *p* = 0.559; Table 1] and no differences in any physiological or engagement metrics (Table 3).

**TABLE 3:** Demographic, engagement metrics and heart rate data for laboratory and remote participants in the screened and screened/matched samples with and without baseline data.

Metric	Screened sample unmatched for age				Screened sample matched for age				Screened sample matched for age with baseline			
	Laboratory	Remote	*U*	*p*	Laboratory	Remote	*U*	*p*	Laboratory	Remote	*U*	*p*
	M(SD)	M(SD)			M(SD)	M(SD)			M(SD)	M(SD)		
Total time on task (s)	366.12 (185.63)	398.92 (195.85)	3166.5	0.212	384.56 (201.27)	408.79 (191.79)	1015.5	0.383	375.63 (153.29)	432.10 (199.48)	489.0	0.280
Mean time per trial (s)	21.54 (10.92)	23.47 (11.52)	3166.5	0.212	22.62 (11.84)	24.05 (11.28)	1015.5	0.383	22.10 (9.02)	25.42 (11.73)	489.0	0.280
Mean engagement^2^	26.52 (15.51)	26.61 (13.79)	3421.5	0.655	27.89 (17.08)	27.24 (13.35)	1095.0	0.776	26.38 (12.26)	28.69 (14.16)	526.0	0.528
N of valid trials	19.25 (0.84)	19.10 (1.04)	3725.5	0.585	19.20 (0.92)	19.02 (1.12)	1206.5	0.568	19.12 (0.91)	19.09 (1.08)	569.0	0.911
Age	22.65 (6.75)	31.75 (9.69)	1272.5	**< 0.001**	24.15 (5.03)	23.69 (2.85)	1100.0	0.803	76.12 (11.19)	72.28 (10.23)	672.0	0.253
Resting heart rate (bpm)	–	–	–	**–**	–	–	–	–	123.44 (77.83)	98.15 (50.68)	672.0	0.253
Heart rate variability (SDNN)	–	–	–	**–**	–	–	–	–	91.77 (45.63)	86.95 (36.43)	593.0	0.860
Heart rate variability (RMSSD)	–	–	–	**–**	–	–	–	–	41.97 (17.32)	41.36 (18.06)	597.0	0.820
Heart rate variability (pNN50)	–	–	–	**–**	–	–	–	–	24.50 (4.69)	23.85 (2.93)	598.0	0.810

^1^Where “engagement” is defined as the number of unique dial positions in a trial, as in Spooner et al. (2024a).

^2^Where “engagement” is defined as the number of unique dial positions in a trial, as in Spooner et al. (2024a). Bold text denotes significance.

## 4 Discussion

### 4.1 Additional refinements

**FIGURE 7:**
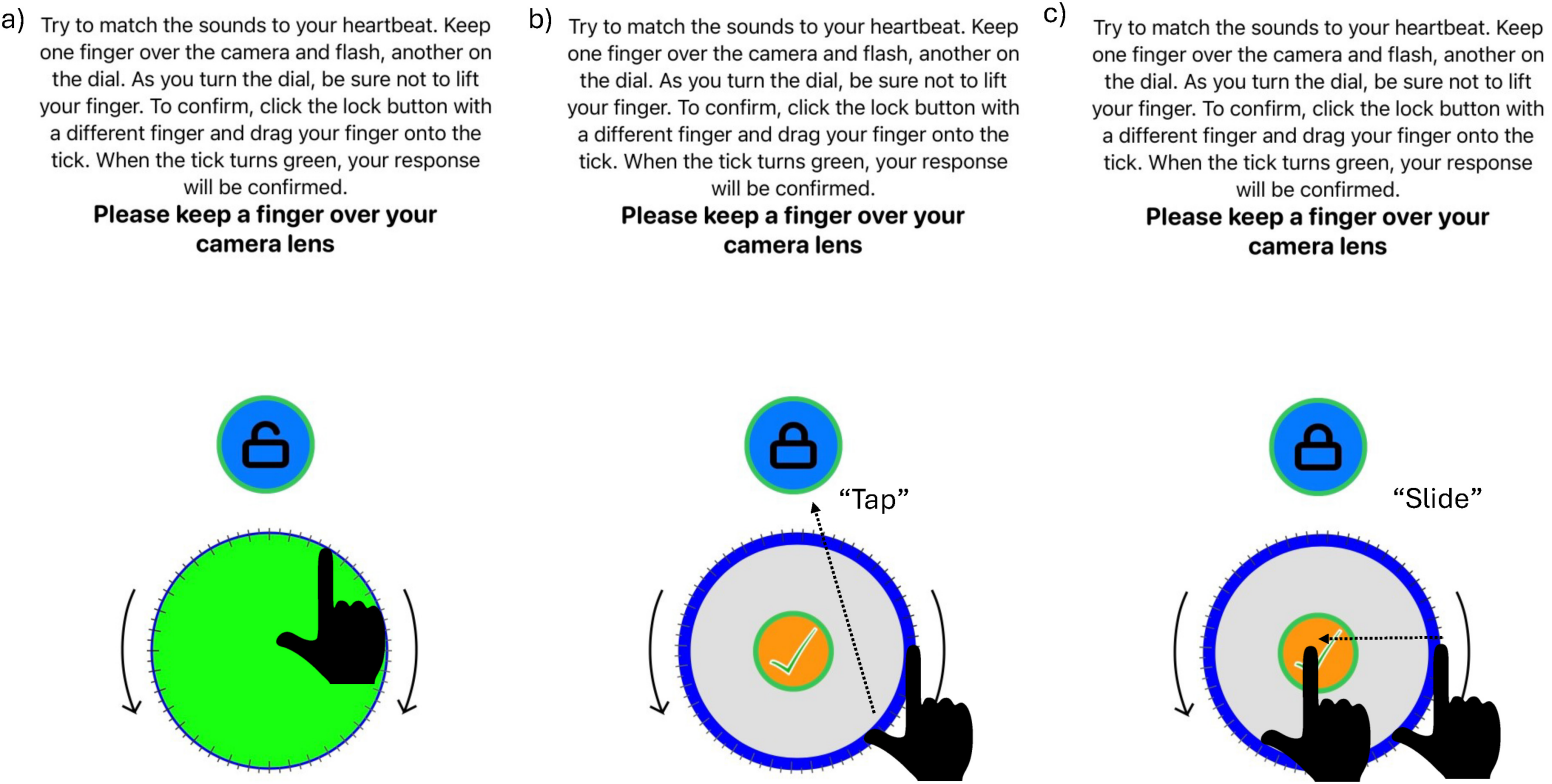
Visualization of the cheat avoidance mechanism. On each trial participants are asked to keep one finger on the dial at all times **(a)**. Once they believe the tones to be synchronous with their heartbeat, they are asked to use a different finger of the same hand to “tap” the lock button **(b)**. To confirm their response, they are then asked to slide their finger from the dial to the tick button **(c)**.

As the PAT has been programmed such that it can be implemented remotely, concerns have been raised that participants may not follow instructions to refrain from checking their pulse, and thus achieve good performance by manually checking their pulse during the task (Desmedt et al., 2023). Whilst recent research has found no evidence that this is the case, as participants perform similarly when the task is administered remotely in an unsupervised fashion and supervised in the laboratory (Spooner et al., 2024a; and section 2.2 above), to discourage participants from checking their pulse we have implemented a new mechanism to discourage “cheating.” In PAT 2.0, participants are asked to place one finger over the camera and flash on the back of their smartphone to enable heartbeats to be recorded. In the original task, participants could conceivably use their other hand to rotate the dial, then remove their hand from the dial to check their pulse, place it back on the dial to make an adjustment, remove it to check their pulse, and so on until they felt the tones were synchronous with their heartbeat. Although this kind of behavior could be easily detected in the time taken on each trial (which does not vary as a function of testing method; see Spooner et al., 2024a), in the revised task participants are required to keep one finger of the other hand on the virtual dial at all times during the trial. When they believe the tones to be synchronous with their heartbeat, they click a “lock” button with another finger of that hand to confirm their response, before dragging the index finger of their responding hand to a confirmation point to submit their response (see Figure 7). Removal of the finger from the dial for longer than 1000 ms results in the trial being aborted and these trials are identified and discarded by the updated analysis script. This trial is then repeated with a new starting phase. Participants are allowed to trigger this “cheat-avoidance” mechanism as many times as required during the practice trials to enable them to become familiar with the task. During the main task, the task continues until a minimum of 20 valid trials have been completed, or the cheat-avoidance is triggered too many times (currently participants can attempt a maximum of 40 trials; when participants breach this threshold their data is discarded due to a failure to follow instructions). Whilst the number of cheat-avoidance trials allowed will depend on the study implementation (e.g., if participants are observed during the task any number will be acceptable as it will be possible to determine whether participants attempted to manually check their pulse), for unobserved remote testing the experimenter may wish to deploy a more conservative criterion to exclude participants depending on the population being studied. This can be achieved by adjusting the parameters in the analysis code to exclude individuals who trigger the cheat-avoidance too frequently. Overall, whilst no mechanism other than supervised administration (which can occur remotely via video call) will fully prevent some participants checking their pulse, existing evidence suggests that this is rare (Spooner et al., 2024a), and the cheat-avoidance mechanism reduces the likelihood further. It should be noted that the cheat avoidance mechanism may be difficult to perform for individuals or groups with decreased hand mobility, and so it may be detrimental to use with these participants, especially if testing is observed and cheating therefore not possible.

### 4.2 Further recommendations

Whilst we have implemented no changes to the total number of task trials, it is worth noting that 20 trials are used in PAT 2.0. This is because simulations of original expected consistency profiles suggested that a great deal of information was gained by increasing trial numbers between 1 and 15 trials, but very little information was gained beyond 20 trials (see Plans et al., 2021; Todd et al., 2024), and later simulations suggest that the same is true for our novel analysis approach (although with 25 trials false negative rates close to 0% are observed even with ultra-high IBI variability using fixed-period scoring). Thus, where trials are excluded by the provided analysis scripts (e.g., because of a lack of participant engagement on a single trial), participants can be retained so long as 15 valid trials are available. Whilst we recommend pre-registering a threshold (e.g., in Spooner et al., 2024a a threshold of 17 valid trials was set), 15 trials are an acceptable minimum that reduces the risk of classifying too many “interoceptive” participants as “non-interoceptive.” The number of trials required can be adjusted by changing this parameter in the provided analysis script.

## 5 Summary and conclusion

This paper reports on refinements to the PAT aiming to optimize measurement of cardiac interoceptive accuracy including: (1) triggering tones from the detection of the heartbeat via the smartphone camera, rather than using an algorithm for predicting the occurrence of the next heartbeat; (2) technical amendments to enable implementation on iPhones with multiple cameras; (3) changes to the instructions; and (4) collection of confidence ratings to improve participant understanding and the utility of confidence ratings for interpretation of results; (5) further practice trials to aid clarity, as well as refinements to the structurally identical screener task; (6) changes to the analysis method to detect phase-based response patterns; (7) a review of the use of continuous scores and important considerations for utilizing the PAT under perturbation conditions (e.g., exercise); (8) a reanalysis of data comparing PAT performance in supervised laboratory testing and unsupervised remote testing using the new scoring method; (9) the implementation of additional measures to discourage participants from checking their pulse during the task, and (10) additional recommendations and clarifications relating to minimum trial numbers.

In addition, this paper outlines several practical recommendations to improve usability of the PAT and interpretation of results. This includes the recommendation for all studies utilizing the PAT to employ the structurally identical screener task to enable it to be determined whether performance can be attributed to difficulties with heartbeat perception (rather than multisensory integration, wider cognitive impairment or otherwise), and recommendations for future work to employ repeated testing to quantify the number of situations and occasions that a participant can perceive their heartbeat given evidence of state effects on cardiac perception. Indeed, we believe that this may provide a more accurate reflection of interoceptive ability than can be captured by testing on one occasion. Whilst further refinements are required—including the development of a lab-based ECG implementation of the PAT, as well as an Android version to maximize accessibility—it is hoped that these improvements should enable those planning to use the PAT to do so effectively, and increase standardization of measurement across studies increasing the degree to which comparisons can be drawn.

## Data Availability

The data analyzed in this study is subject to the following licenses/restrictions: The data that support the findings of this study are available from the corresponding author upon reasonable request. Requests to access these datasets should be directed to Jennifer Murphy (jennifer.murphy@surrey.ac.uk).

## Footnotes

1 Note that variable heart rate over trials is not the same as heart rate variability as classically calculated. Here we are referring to variations in the inter-beat-interval over trials, rather than variation from one heartbeat to the next.
