# Supplementary material for: Refinements of the phase adjustment task (PAT 2.0)
Source: Front Psychol. 2026 May 29;17:1677186. doi: 10.3389/fpsyg.2026.1677186 (PMC13261906; doi:10.3389/fpsyg.2026.1677186)
Supplement: Supplementary file 1 [file Data_Sheet_1.PDF]

Supplement

S1: Changes to the instructions PAT 1.0 to PAT 2.0

Note: Removals are indicated with a ~~strikethrough~~. Additions are highlighted in yellow.

How well can you match a sound with your heartbeat? Let's find out!

1. First, find a quiet place where you can comfortably sit upright ~~with your earphones on~~ for around 25 minutes. Please don't wear earphones. We will begin by capturing a couple of minutes of your heart rate, to get a sense for how your heart does its thing.
2. Get ready to check your heartbeat
    a. We will shortly turn on the LED Flash and camera on this phone, and will use it to take your heart rate. Please place your index finger over both the camera and flash and try to turn the rectangle red.

[Make the rectangle red]

3. Please unmute your device and make sure you can hear the tone before continuing.

4. Ok, thanks! In the main task, you will be asked to place your finger on the flash and camera (on the back) so that the app can read your heartbeat. Once your finger is in position, you will hear a series of sounds. Each sound actually represents one of your own heartbeats!
5. It might seem like there is a delay between the sounds and the heartbeats you feel. Play the video below to hear an example!

[Video heartbeat sounds out of sync]

6. In order to fix the delay, you will be asked to move a dial until the sounds are in sync with your heartbeats. Play the video below to hear an example!

[Video heartbeat sounds in sync]

7. ~~If you move the dial to the right, the delay between the heartbeat and the sound will get longer; if you move it to the left, the delay will get shorter.~~ Your objective is to find the point on the dial where the heartbeat and sound is in sync, by turning the dial left or right. There will be 20 trials in total.
8. Want to know what this looks like? Press "continue" to watch a short tutorial.

[Instructions video]

~~After you have matched the sound with your heartbeat, you will be asked how sure you are about the answer you gave. Press "confirm" then "continue" to start the following trial. In this task, there will be 20 trials in total.~~

~~[EXAMPLE OF THE CONFIDENCE SCALE]~~

9. ~~You~~ People can feel their heartbeat in different places in their body, such as their chest or fingers. ~~You will be asked to indicate where you felt your heartbeat on a body map (like the one below) once every 5 trials~~. Where do you currently feel your heartbeat most clearly? Please show us on the body map below. You can choose any of the highlighted body parts ~~or~~

~~you can select "nowhere" if you haven't felt your heartbeat in any particular place~~. If you can't feel it at all, press "nowhere". PLEASE only use heartbeats you feel in that location throughout all trials.

[Insert body map]

10. Don't worry if you find this task difficult, people are sometimes better than they think they are! For the duration of this task, please do not actively try to feel your pulse with your hand; we are only interested in what you feel inside! You might feel your heartbeat in various bodily locations. Just make sure you pick one and stick to using that one during the task. When you are ready to start, please sit comfortably upright ~~with your earphones on~~ and press "continue".

11. You will now get a chance to do some practice trials where we will ask you to try and match two beeps together. You can move your finger to turn the dial (but you are not allowed to lift it) once you have started. To confirm your response click the lock button with a different finger and then drag your finger onto the tick in the middle of the dial and wait for it to turn green. After you hold this for 2 seconds your response will be submitted and the next trial will start.

12. PRACTICE. Move the dial until the tones happen at the same time. Please press confirm when you are done. To confirm your response, click the lock button with a different finger and then drag your finger onto the tick on the middle of the dial and wait for it to turn green. After you hold this for 2 seconds your response will be submitted and the next trial will start.
    a. Please keep a finger over your camera lens.
    b. Keep your finger on the dial until you have finished turning it

13. Now it is time for the main practice. Focus on feeling your heartbeat and try to match the sounds to your own heartbeat. Remember to keep one finger over the camera and flash and your other finger on the dial. You can move your finger to turn the dial (but you are not allowed to lift it) once you have started. To confirm your response, click the lock button with a different finger and then drag your finger onto the tick on the middle of the dial and wait for it to turn green. After you hold this for 2 seconds your response will be submitted and the next trial will start. If you accidentally lift it too early you can have another go, but you will only get 2 retries.

14. MAIN PRACTICE: Try to match the sounds to your heartbeat. Keep one finger over the camera and flash, another on the dial. As you turn the dial, be sure not to lift your finger. To confirm, click the lock button with a different finger and drag your finger onto the tick. When the tick turns green, your response will be confirmed.

    a. Please keep a finger over your camera lens.
    b. Keep your finger on the dial until you have finished turning it

15. MAIN TASK: Now it is time for the main task. Focus on feeling your heartbeat and try to match the sounds to your own heartbeat. Remember to keep one finger over the camera and flash and your other finger on the dial. You can move your finger to turn the dial (but you are not allowed to lift it) once you have started. To confirm your response, click the lock button with a different finger and then drag your finger onto the tick on the middle of the dial and wait for it to turn green. After you hold this for 2 seconds your response will be submitted and the next trial will start. If you accidentally lift it too early you can have another go, but you will only get 2 retries.

During trials: ~~Move the dial until the tone matches your heart-beat, to the best of your perception. Please press confirm when you are done.~~ Try to match the sounds to your heartbeat. Keep one finger over the camera and flash, another on the dial. As you turn the dial, be sure not to lift your finger. To confirm, click the lock button with a different finger and drag your finger onto the tick. When the tick turns green, your response will be confirmed.

At the end of the task…

16. How much do you agree with the following statement? I am confident I could feel my heartbeat during the heartbeat task. (response options from Strongly disagree to Strongly agree on a 7-point scale).

17. How much do you agree with the following statement? I am confident I could match the tones to my heartbeat during the task. (response options from Strongly disagree to Strongly agree on a 7-point scale).

18. How many locations on your body did you use to feel your heartbeat during the task?
    1. Please indicate where they are on the body map below.

[Body map]
